# The E1B19K Oncoprotein Complexes with Beclin 1 to Regulate Autophagy in Adenovirus-Infected Cells

**DOI:** 10.1371/journal.pone.0029467

**Published:** 2011-12-29

**Authors:** Sujan Piya, Erin J. White, Sarah R. Klein, Hong Jiang, Timothy J. McDonnell, Candelaria Gomez-Manzano, Juan Fueyo

**Affiliations:** 1 Department of Neuro-Oncology, The University of Texas MD Anderson Cancer Center, Houston, Texas, United States of America; 2 Department of Hematopathology, The University of Texas MD Anderson Cancer Center, Houston, Texas, United States of America; 3 Department of Genetics, The University of Texas MD Anderson Cancer Center, Houston, Texas, United States of America; Istituto Nazionale per le Malattie Infettive, Italy

## Abstract

The mechanisms underlying adenovirus-mediated autophagy are currently unknown. Recently, members of the Bcl-2 protein family have been associated with autophagy. It was also reported that the Bcl-2 homology-3 (BH3) domain encompassed by both Beclin 1 and Bcl-2-like proteins is essential for their pro-autophagy or anti-autophagy functions. Here, we report for the first time that E1B19K, the adenovirus BH3 domain protein, interacts with Beclin 1 to initiate autophagy. Using immunoprecipitation assays we showed that expression of E1B19K in the host cell disrupted the physical interactions between Beclin 1 and Bcl-2 proteins. The displacement of Bcl-2 was coincident with the recruitment of PI3KC3 to the Beclin 1/E1B19K complexes. As a result of the changes in the components of the Beclin 1 interactome, there was activation of PI3KC3, as showed by the identification of PI3K-mediated lipid phosphorylation, and subsequent formation of autophagosomes. Importantly, the BH3 functional domain of E1B19K protein was required for the heterodimerization with Beclin 1. We also showed that transfer of E1B19K was sufficient to trigger autophagy in cancer cells. Consistent with these data, mutant adenoviruses encompassing a deletion of the *E1B19K* gene produced a marked deficiency in the capability of the virus to induce autophagy as showed by examining the lipidation and cleavage of LC3-I as well as the subcellular localization of LC3-II, the decrease in the levels of p62, and the formation of autophagosomes. Our work offers new information on the mechanisms of action of the adenoviral E1B19K protein as partner of Beclin 1 and positive regulator of autophagy.

## Introduction

Accumulating evidence shows that cells infected with adenoviruses undergo autophagy [1], [2], [3], [4]. Because autophagy is part of the innate immunity against pathogens, bacteria and viruses have developed strategies to halt [5], [6] or subvert the autophagy process in the host cell to enhance their replication [7] or as an efficient mechanism to release the new progeny of virus [5], [6], [8].

We have recently shown that autophagy is used by adenoviruses to efficiently induce cell lyses [8], but the molecular mechanisms underlying the regulation of autophagy in adenovirus-infected cells are unknown. Because previous studies showed that Bcl-2 protein interacts with Beclin 1 [9], and herpes virus Bcl-2 like proteins modify autophagy in the host cells [6], [10], we hypothesized that the E1B19K adenovirus protein, a Bcl-2 homolog [11], heterodimerizes with Beclin 1 to regulate autophagy by modulating the interaction of Beclin 1 with PI3KC3 [9].

This hypothesis is also supported by several reports demonstrating that BH3 domain proteins in addition to modulate apoptosis, are involved in the regulation of autophagy [12]. In this regard, two members of this family of proteins, Bcl-2 and Bcl-_XL_, were identified as interactors of Beclin 1 [6], [10], [13], [14], [15]. In addition of these negative regulators of autophagy several members of the BH3 family were reported to function as autophagy inducers including the adenovirus-E1B19K/Bcl-2-interacting-protein (BNIP3) [16], BAD and EGL-1 [17]). It is important to mention that BNIP3 protein interacts with E1B19K [18].

E1B19K is thought to be a “virus Bcl-2” homologue because encompasses a functional BH3 domain [19], [20], [21], [22] and interacts with Bax, but not with Bcl-2, to block apoptosis [23]. However, the role of E1B19K in the regulation of autophagy has not been elucidated.

In this study, we showed that during an adenoviral infection, E1B19K interacts with the Beclin 1/PI3KC3 complexes in the host cells resulting (opposite from what is described for other viral Bcl-2 proteins [6], [10]) in the activation of PI3KC3 activity, and the subsequent initiation of autophagy. We also observed that the functional BH3 domain of E1B19K is required for this interaction. Interestingly, while expression of E1B19K is sufficient to trigger autophagy. In this study, we therefore provide new information on the cellular roles of E1B19K including interactions with Beclin 1 and regulation of autophagy.

## Results and Discussion

**Figure 1:**
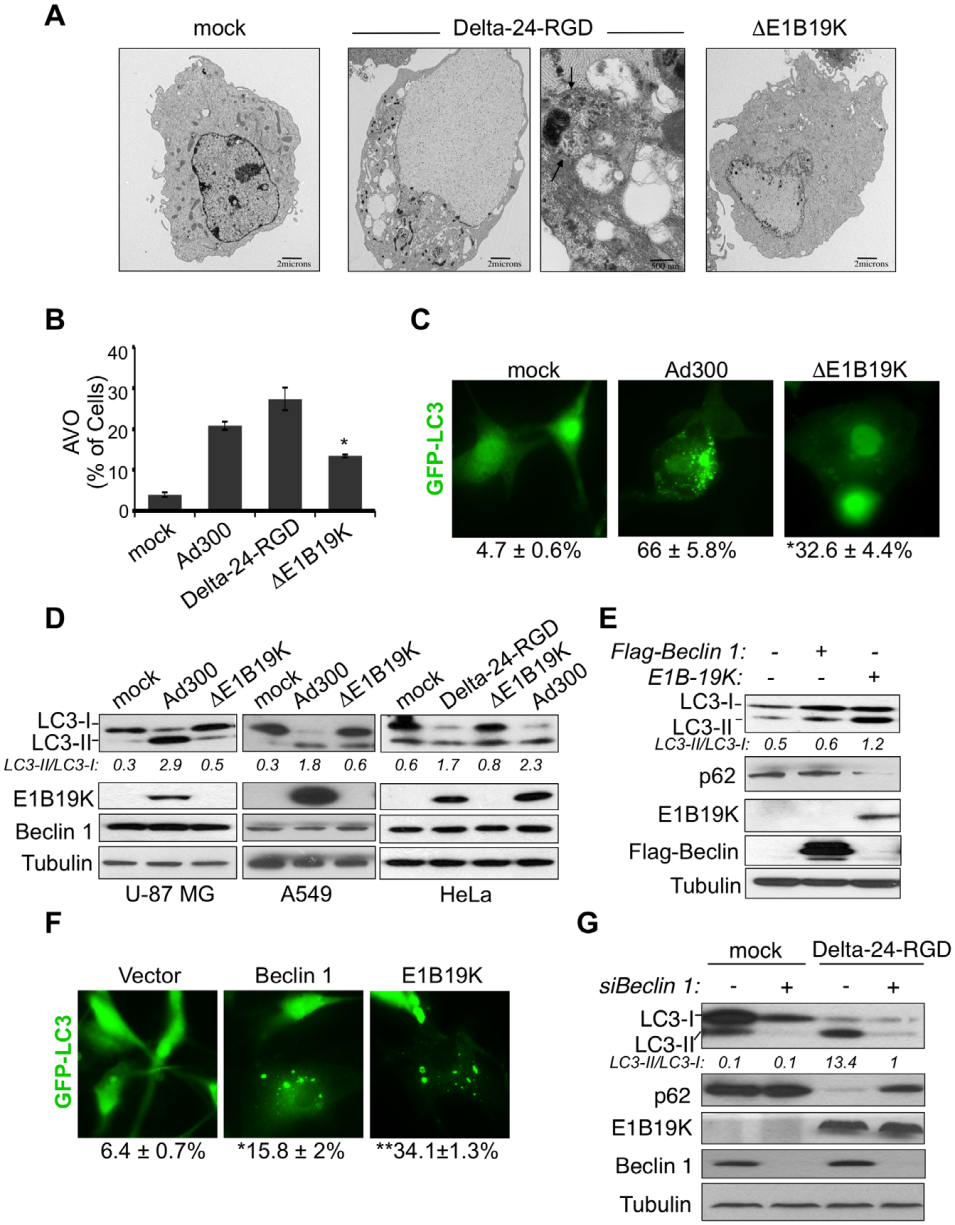
The E1B19K protein plays an essential role in adenovirus-mediated autophagy. A. Representative electron micrographs of U-87 MG glioma cells infected with Delta-24-RGD or ΔE1B19K. Cells infected with Delta-24-RGD exhibited autophagolysosomal formation, as evidenced by double membrane organelles and cytoplasmic contain (arrows). B. Quantification of AVO after infection of U-87 MG cells with the indicated adenoviruses. Data are represented as mean ± SD of three independent experiments. *P<0.01 versus Ad300-infected cells (two-tailed Student's t-test). C. Deletion of E1BK jeopardizes adenovirus-associated autophagy, as assessed by a significant decrease in the LC3 membrane localization. U87.GFP-LC3 cells were infected with Ad300 or ΔE1B19K (50 MOIs; 48 h). Shown are representative fluorescence images (Zeiss AxioImager 373), and mean ± SEM of three independent experiments of percentage of cells displaying GFP-LC3-positive puncta. *P<0.001 versus Ad300-infected cells (two-tailed Student's t-test). D. Representative immunoblots from a panel of cancer cells after mock-infection or infection with the indicated adenoviruses show a decrease in the LC3-I to LC3-II conversion of cells infected with ΔE1B19K (indicated as LC3-II/LC3-I ratio). E. U87MG.GFP-LC3 cells were transfected with pcDNA.Flag, pcDNA.Flag-Beclin 1, or pcDNA.E1B19K, and 48 h later whole cell lysates were subjected to Western blot analyses to analyze the biochemical markers of autophagy: LC3-I to LC3-II conversion (indicated as LC3-II/LC3-I ratio) and p62 degradation. F. Representative fluorescence images of U-87 MG.GFP-LC3 after the indicated treatments are shown. Percentage of puncta-positive cells were quantified and data are represented as mean ± SEM from three independent experiments, from a minimum of 100 total cells per test. *P<0.01 and ** *P*<0.001 versus the mock group (two-tailed Student's t-test). G. HeLa cells were transfected with siBeclin or siControl and 24 h later, cells were mock- or Delta-24-RGD-infected (10 MOIs). After 48 h, cells were subjected to Western blot analysis to analyze LC3-I to LC3-II conversion (indicated as LC3-II/LC3-I ratio) and p62 degradation. E1B19K and Beclin 1 levels are shown, and tubulin levels were used as loading control.

Recent work has shown that the intracellular adenovirus life cycle results in the induction of autophagy [1], [2]. Here, we hypothesized that E1B19K heterodimerizes with Beclin 1 and, by doing so, positively regulates autophagy. To test this hypothesis, we analyzed biochemical markers and cellular events to assess autophagy in human glioma cells infected with wild-type adenovirus, the E1A-mutant adenovirus Delta-24-RGD [24], or an E1B19K-deleted adenovirus (ΔE1B19K). As expected, infection with wild-type or Delta-24-RGD adenoviruses induced productive autophagy as assessed by electron microscopy, acidic vesicular organelle (AVO) formation, green fluorescent protein (GFP)-LC3 localization on the membrane of autophagosomes, and cleavage and lipidation of the LC3-I protein (Fig. 1A–D). Interestingly, autophagy was dramatically attenuated in cells infected with ΔE1B19K (Fig. 1A–D), strongly suggesting that E1B19K plays a role in adenovirus-mediated autophagy. The decrease in autophagy observed in the ΔE1B19K-infected cells was consistent with data from a recent study showing that E1B-deleted adenoviruses lacking the expression of E1B55K and E1B19K are impaired for the induction of autophagy [18], [25].

While infected cells are the natural environments to characterize the phenomenon of adenoviral infection, it is difficult to isolate the role of a single viral protein in this extremely complex scenario. For that reason, we transfected cells with E1B19K and observed that transfer of the viral protein sufficed to trigger autophagy (Fig. 1E and 1F). In addition, silencing of Beclin 1 was sufficient to render cells resistant to adenovirus-mediated autophagy (Fig. 1G), supporting our hypothesis that both proteins cooperate to induce autophagy.

**Figure 2:**
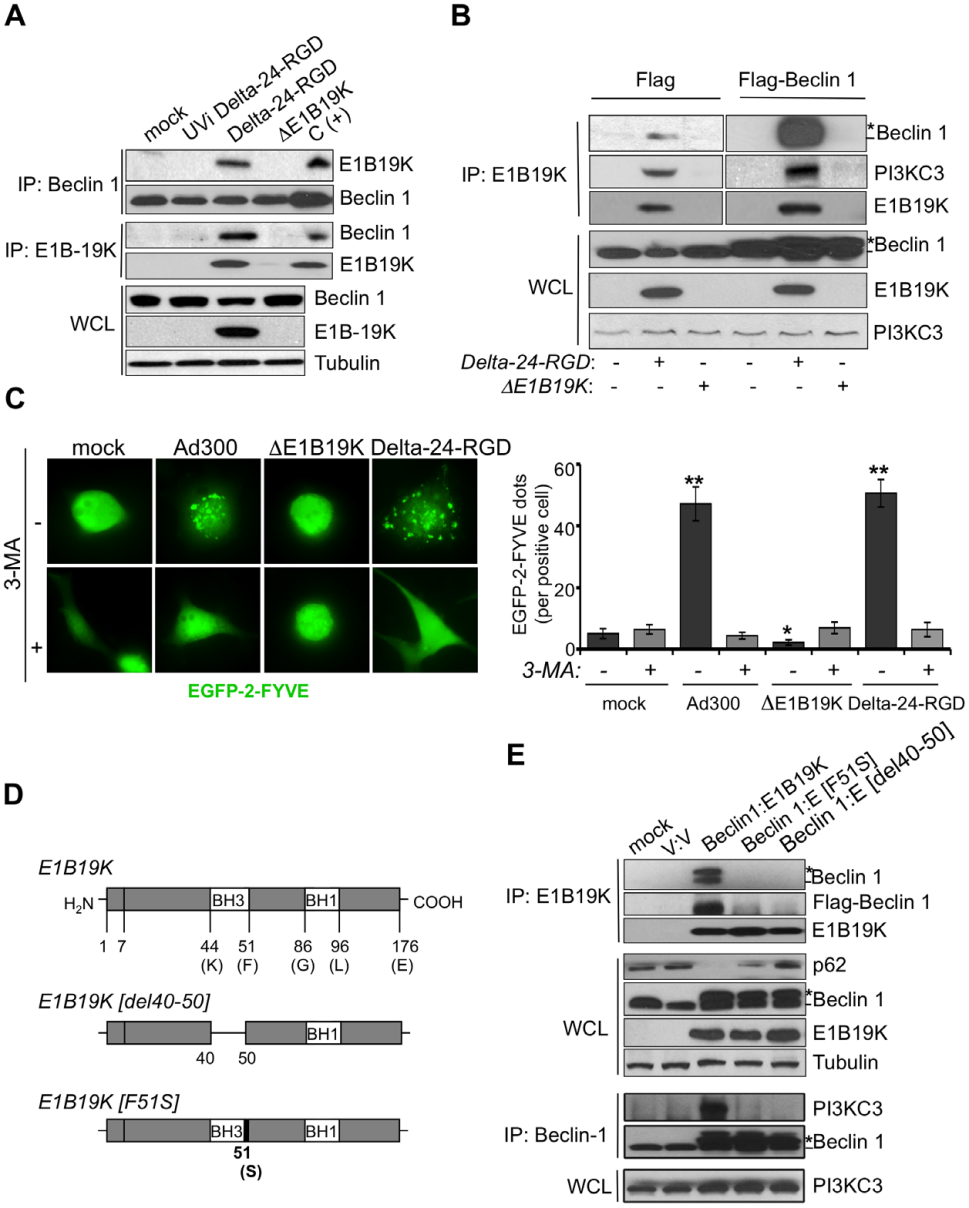
E1B19K interacts with Beclin1 leading to activation of PI3KC3. A. HeLa cells were infected with ultraviolet-inactivated (UVi) Delta-24-RGD, Delta-24-RGD, or ΔE1B19K (10 MOIs; 24 h). Whole cell lysate was analyzed by immunoprecipation for the presence of E1B19K/Beclin complexes. WCL, whole-cell lysates; IP, immunoprecipitation. B. Cells were transfected with pcDNA.Flag or pcDNA.Flag-Beclin 1, as described in Figure 1E. 24 h later cells were then infected with the indicated virus (10 MOIs, 24 h). Whole-cell lysates were immunoprecipitated with anti-E1B19K. *, indicates the detection of Flag-Beclin 1. C. HeLa cells were transfected with EGFP-2xFYVE and 24 h after cells were infected with the indicated adenoviruses (10 MOIs, 24 h) or were mock-transfected, in combination with 3-MA (3 mM) or vehicle. PI3KC3 activity was observed as the presence of a fluorescence punctate pattern in the cells (left panel). The number of EGFP-positive dots per positive cells is represented as mean ± 95% CI (right panel). * *P*<0.001 versus Ad300 and Delta-24-RGD-treated groups and ** *P*<0.001 versus mock group (two-tailed Student's t-test). D, E. HeLa cells were transiently transfected with plasmids expressing wild-type E1B19K, or mutant forms of E1B19K (F51S and del40-50) [21] (D) in combination with plasmids expressing wild-type Beclin 1. 48 h later cell lysates were collected and analyzed for the presence of E1B19K/Beclin 1 and PI3KC3/Beclin 1 complexes (E). WCL, whole cell lysates; IP, immunoprecipitation; *indicated the detection of the exogenous Flag-Beclin 1.

We next showed that E1B19K was part of the Beclin 1 interactome in adenovirus-infected cells (Fig. 2A and 2B). Because the physical association of Beclin 1 with PI3KC3 is important for the membrane localization and function of PI3KC3 [26], we were interested in ascertaining whether E1B19K is an interactor of the Beclin-1-PI3KC3 complex. To this end, Beclin 1-transfected cells were infected with Delta-24-RGD or ΔE1B19K adenovirus, and then cell lysates were immunoprecipitated with either E1B19K or Beclin 1. Interestingly, E1B19K coimmunoprecipitated with both Beclin 1 (Fig. 2A and 2B) and PI3KC3 (Fig. 2B). This protein association suggested PI3KC3 activation, which we confirmed using a construct encompassing the GFP-tagged double FYVE finger of the Hrs protein, which specifically binds to PI(3)P and allows for the identification of PI3K-mediated lipid phosphorylation [27]. As a result of PI3KC3 increased activity, PI(3)P production was dramatically and significantly higher in wild-type adenovirus- and Delta-24-RGD-infected HeLa cells than in cells infected with ΔE1B19K (Fig. 2C).

Because Beclin 1 interacts with Bcl-2 and Bcl-2-related proteins through their BH3 domain [9], [15], we asked whether the BH3 domain of E1B19K is also required for the interaction. To this end, we generated two mutant E1B19K proteins–one in which we deleted the majority of the BH3 domain (E1B19K [del40-50]) and one in which we deleted a single amino acid that is essential for protein-protein interaction (E1B19K [F51S]) [21] (Fig. 2D). Transfer of these mutants to cancer cells showed that deletions in the BH3 motif abrogated the ability of E1B19K to heterodimerize with Beclin 1 (Fig. 2E).

**Figure 3:**
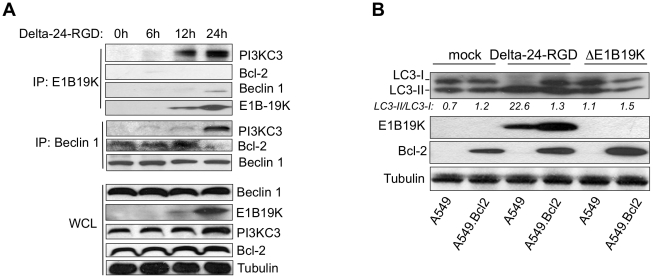
E1B19K competes with Bcl-2 in the Beclin 1 interactome. A. HeLa cells were infected with Delta-24-RGD (10 MOIs) and whole-cell lysates collected at different times points after infection. Whole cell lysates were immunoprecipitated with anti-E1B19K or anti-Beclin 1. WCL, whole cell lysates; IP, immunoprecipitation. B. A549 parental and A549.Bcl2 were mock-treated or infected with Delta-24-RGD or ΔE1B19K (20 MOIs; 48 h). Western blot analysis was performed to analyze LC3-I conversion to LC3-II (indicated as LC3-II/LC3-I ratio).

**Figure 4:**
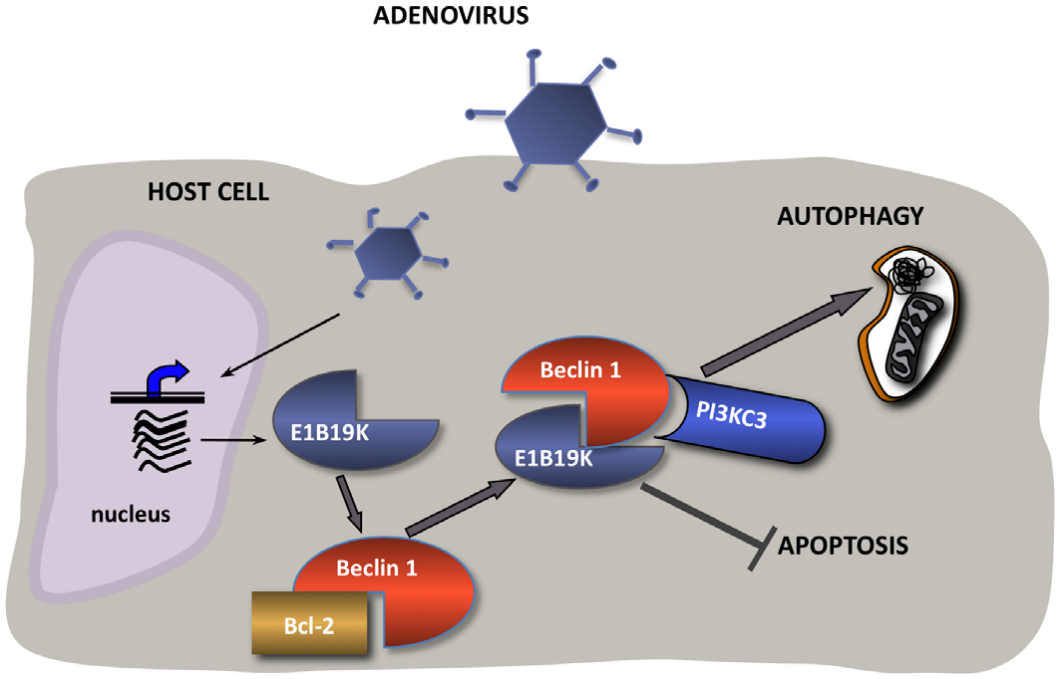
Proposed model of the mechanism underlying the regulation of autophagy by E1B19K. After adenovirus internalization early genes are expressed and E1B19K protein accumulates in the host cell. We describe here that, among other roles, E1B19K will compete with Bcl-2 to form part of the Beclin1 interactome. Eventually, E1B19K expression results in the displacement of Bcl-2 from the Beclin 1 complex. The removal of Bcl-2 (a negative regulator of autophagy) and the integration of E1B19K in the Beclin 1 complex favors the interactions of Beclin 1 and PI3KCIII what in turns results in the formation of autophagosomes and autophagy.

There is experimental evidence that Bcl-2 downregulates autophagy by inhibiting the formation of the Beclin 1-PI3KC3 complex [9]. For that reason, we hypothesized that Bcl-2 and E1B19K were competing to bind Beclin 1 in adenovirus-infected cells. A corollary of this hypothesis was the prediction that Bcl-2 and E1B19K would have opposite effect on the regulation of autophagy. Coimmunoprecipitation analyses of the E1B19K-Beclin 1 complex revealed that presence of PI3KC3 or Bcl-2 in the Beclin 1 complex were mutually exclusive. Thus, Bcl-2 was part of the Beclin 1 complex in non-infected cells, but after 12 h Bcl-2 did not interact with Beclin 1 (Fig. 3A). Accordingly, PI3KC3 was not detected in the E1B19K-Beclin 1 complex before 12 h, but its recruitment to the complex reached a remarkably level at 24 h (Fig. 3A). Collectively, our data provided strong evidence that one of the mechanisms by which E1B19K promoted autophagy involved antagonizing Bcl-2 through direct competition to heterodimerize with Beclin 1. Consistent with this, Bcl-2-transfected cells were more resistant to wild-type adenovirus-induced autophagy than the parental cells (Fig. 3B). These observations support a model in which E1B19K replaces Bcl-2 in the Beclin 1 complex activating autophagy (Fig. 4).

This is the first report documenting the physical interaction of an adenoviral protein with a key autophagy regulator, and suggests that adenoviruses can be used to ascertain the regulatory mechanisms of autophagy in eukaryotic cells. Our data demonstrate that in adenovirus-infected cells, dynamic intra-complex competitions occur in the Beclin 1 interactome, and that viral proteins compete with cellular proteins to bind to the BH3 domain of Beclin 1 to regulate autophagy. These results are congruent with the tenet that BH3 proteins modulate autophagy through their interactions with Beclin 1 [9], [14], [17]. However, the BH3 domain is conserved in both anti-autophagy (Bcl-2 and Bcl-_XL_) and pro-autophagy (BNIP3) [16], BAD and EGL-1 [17]) proteins. We found that the function of E1B19K is similar to that of BNIP3, which induces autophagy by disrupting the Beclin 1-Bcl-2 complex [16]. Because other BCl-2-like viral proteins suppress autophagy during viral infection [6], [10], our data suggest that viruses express BH3 proteins whose roles are similar to either Bcl-2 or BINP3 to respectively suppress or activate autophagy.

It has been shown that E1B19K functions as an antiapoptotic factor in the host cells [21], [23] and now we showed that E1B19K is key in the positive regulation of autophagy. Both functions of E1B19K are key and complementary and suggest that E1B19K plays a fundamental role in maintaining the cell alive to allow a complete and successful viral cycle. The interaction of E1B19K with Beclin1 explains its pro-autophagy function, and it is through the interaction with members of the Bcl2 family of proteins that E1B19K regulates apoptosis [18], [19], [20], [21], [22], [23]. The simplicity of the system that allows an adenoviral protein to interact using the same domain (BH3) with autophagy- and apoptosis-related cellular proteins is consistent with the required economy of viral material and the need to exert an enormous variety of functions during the virus-host interactions [28]. Deletion of E1B19K did not completely suppressed autophagy, indicating that other factors may be relevant in the regulation of adenovirus-mediated autophagy. We cannot rule out that ER stress -due to accumulation of viral proteins- does not play a role in the adenovirus-mediated autophagy process [29]. Nevertheless, our data showed a new function of E1B19K that complements and expands previous knowledge about this adenoviral protein.

In summary, we have identified a molecular mechanism that underlies the ability of E1B19K to activate autophagy. Autophagy is observed after adenoviral infection *in vitro* and *in vivo* during an efficacious adenoviral replication [1], [2] and our data indicate that human adenoviruses might have evolved their own mechanisms to trigger autophagy, and that autophagy might facilitate the cellular landscape for viral replication and/or cell lysis [30]. Our study also suggests that a comprehensive and mechanistic analysis of the autophagy targets of adenoviral oncoproteins should result in a better understanding of the cellular pathways that are dysfunctional in virus-associated cancers [31] and perhaps offer new insights on the regulation of these networks in the rest of cancers. Further studies are required to establish whether the role that E1B19K and other viral Bcl-2 proteins play in the regulation of autophagy and apoptosis are independent functions or, on the contrary, are coordinated and cooperate to induce malignant transformation. Further examination of the functional relationship between viral proteins and autophagy could provide new information about the potential role that virus-induced autophagy, in particular, and autophagy pathways, in general, play in oncogenesis [32].

## Materials and Methods

### Cell cultures

A549, HeLa, HCT116, HEK293, U-87 MG, and U-251 MG cell lines were obtained from ATCC and maintained in DMEM/F12 or McCoy's 5A medium (HCT116) (Gibco, Invitrogen). Bcl-2-overexpressing A549 cultures (A549.Bcl2) were generated by cloning a 1.8-kb fragment of the Bcl-2 cDNA containing the open reading frame into an expression plasmid (pSFFV-neo). Stable empty vector control cells and Bcl-2 over-expressing clones were generated using standard gene transfer techniques and selection in Geneticin (Life Technologies). Generation of U-87 MG cells stably expressing GFP-LC3, U87.GFP-LC3, were reported elsewhere [33].

### Reagents

5-flurouracil (5-FU) and 3-MA were purchased from Aldrich-Sigma.

### Quantification of acidic vesicle organelle (AVO) formation

Acridine orange staining of the cells was conducted as described previously [1]. Briefly, 1.0 µg/ml acridine orange was added to the cell culture, and the cells were incubated for 15 minutes. Stained cells were then analyzed by flow cytometry using a FACScan cytometer and CellQuest software (Becton Dickinson, San Jose, CA, USA). Red (FL3-H channel) fluorescence emissions from 10^4^ cells were analyzed, and quantified as percentage of cells containing AVO.

### Antibodies

Tubulin (Chemicon); Beclin-1, LC3 (Cell Signaling Technology); Bcl-2, p62 (Santa Cruz Biotechnology, Inc.); PI3KCIII, Flag tagged (Aldrich-Sigma); E1B19K (Calbiochem); horseradish peroxidase (HRP)-conjugated goat anti-rabbit and anti-mouse IgG (Santa Cruz Biotechnology).

### Adenoviruses

A mutant form of adenovirus that does not express E1B19K (ΔE1B19K) was produced. Briefly, site-directed mutagenesis (Stratagene) was performed per the manufacturer's instructions on pXC1 (Microbix) to insert two stop codons at the beginning of the reading frame of E1B19K, using the primer pair E1B19K sense-5′ atctgacctcatggaggcttgatagtgtttggaagatttttctg and anti-sense-5′ cagaaaaatcttccaaacactatc aagcctccatgaggtcagat. HEK-293 cells were cotransfected with the mutated pXC1 and pBHG10 adenoviral plasmids (Microbix) using FuGENE 6 transfection reagent (Roche Diagnostics). ΔE1B19K was amplified in HEK-293 cells and purified using cesium chloride gradient centrifugation. Wild-type adenovirus Ad300 and Delta-24-RGD, and infection conditions have been previously reported [24].

### Plasmids

pcDNA3.1(+) (Invitrogen), pcDNA.Flag (generous gift of Dr. Z. Lu, MDACC), pcDNA.Flag-Beclin-1, was generated by inserting Beclin-1 downstream of Flag sequence. pcDNA.E1B19K was generated by PCR-based subcloning of E1B19K sequence form pXC1 (primer pair: 5′-cgggatccatggaggcttgggagtgtttggaa gat-3′ and 5′-gccggcctggaccctcgggaatgagaattccg-3′).

### GFP-LC3 detection

U87MG.GFP-LC3 cells [34] were analyzed by using a fluorescence microscope, and the percentage of cells displaying GFP-LC3-positive puncta was quantified by counting five random fields in three independent experiments.

### Western Blotting

Cells were lysed in radioimmunoprecipitation assay buffer (1% NP-40, 0.5% sodium deoxycholate, 0.1% sodium dodecyl sulfate (SDS), 50 mM Tris-Cl [pH 7.5], and 150 mM NaCl) in the presence of 1× protease cocktail inhibitor. Soluble lysates were subjected to SDS-polyacrylamide gel electrophoresis (PAGE) and transferred to polyvinylidene fluoride membranes (Bio-Rad). Membranes were probed with specific antibodies against: LC3 and Beclin 1 (Cell Signaling Technology), E1B19K (Calbiochem), p62 and Bcl-2 (Santa Cruz Biotechnology, Inc.), PI3KC3 and Flag tagged (Aldrich-Sigma), and tubulin (Chemicon). Immobilon Western chemiluminescent HRP substrate (Millipore) was used for visualization.

### Immunoprecipation

Cultures were collected and lysed with immunoprecipitation lysis buffer (100 mM NaCl, 50 mM Tris-HCl [pH 7.4], 2 mM ethylenediaminetetraacetic acid, 10% glycerol, and 1% IgePal CA-630) in the presence of 1× protease cocktail inhibitor. The lysates were pre-cleared with protein A agarose beads (Millipore). Anti-Beclin 1 or anti-E1B19K antibodies (2–4 µg) were used to pulled down the protein complexes overnight at 4°C, and then complexes were immobilized with protein A and protein G (1∶1) agarose beads. After beads were washed three times in immunoprecipitation lysis buffer, proteins were eluted in SDS loading buffer by heating at 90°C for 10 min. The proteins were separated by the use of SDS-PAGE and probed with specific antibodies as describe in the Immoblotting section.

### siRNA transfection

Cells were transfected with siRNA specific for Beclin 1, siBeclin 1, or scramble siRNA, siControl (2 nM; Santa Cruz Biotechnology, Inc.) as previously described [33].

### PI3KC3 Assay

HeLa cells were transfected with EGFP-2FYVE (GE Healthcare), as a class III phosphatidyl inositol 3-kinase (PI3-kinase) cellular sensor, using Effectene transfection reagent (Qiagen) according to the manufacturer's protocol. The number of GFP-FYVE dots per cell was quantified by counting five random fields and at least 100 cells from three independent experiments.

### Statistical Analysis

For quantitative data analysis, the results were ploted as the mean ± SEM. Statistical significance was determined using Student's t tests.
